# Impact of Oral Mesenchymal Stem Cells Applications as a Promising Therapeutic Target in the Therapy of Periodontal Disease

**DOI:** 10.3390/ijms232113419

**Published:** 2022-11-03

**Authors:** Mariacristina Amato, Simona Santonocito, Gaia Viglianisi, Marco Tatullo, Gaetano Isola

**Affiliations:** 1 Department of General Surgery and Surgical-Medical Specialties, School of Dentistry, University of Catania, 95124 Catania, Italy; 2 Department of Basic Medical Sciences, Neurosciences and Sense Organs, University of Bari, 70122 Bari, Italy

**Keywords:** periodontitis, stem cells, oral mesenchymal, target therapies, periodontal regeneration, bone healing, periodontal ligament

## Abstract

Periodontal disease is a chronic inflammatory condition affecting about 20–50% of people, worldwide, and manifesting clinically through the detection of gingival inflammation, clinical attachment loss, radiographically assessed resorption of alveolar bone, gingival bleeding upon probing, teeth mobility and their potential loss at advanced stages. It is characterized by a multifactorial etiology, including an imbalance of the oral microbiota, mechanical stress and systemic diseases such as diabetes mellitus. The current standard treatments for periodontitis include eliminating the microbial pathogens and applying biomaterials to treat the bone defects. However, periodontal tissue regeneration via a process consistent with the natural tissue formation process has not yet been achieved. Developmental biology studies state that periodontal tissue is composed of neural crest-derived ectomesenchyme. The aim of this review is to discuss the clinical utility of stem cells in periodontal regeneration by reviewing the relevant literature that assesses the periodontal-regenerative potential of stem cells.

## 1. Introduction

Periodontitis is a chronic multifactorial inflammatory disease that is associated with dysbiotic plaque biofilms causing damage to supporting tooth tissues, including the periodontal ligament, cementum and the alveolar bone [1,2,3]. It is manifested by gingival inflammation, clinical attachment level (CAL) loss, radiographic alveolar bone loss, periodontal pocketing, gingival bleeding and tooth mobility [2,3,4]. It is prevalent worldwide, so its treatment is a common interest among clinicians, representing a central point in scientific research. It is characterized by the imbalance of the oral microbiota, which is a necessary but not sufficient factor to cause periodontal disease, in fact, on the other side, to make its development possible, it is necessary to have a genetic predisposition too. It leads to an aberrant inflammatory response that causes damage to the tooth-supporting tissues. Other factors may be included in its development: systemic conditions such as diabetes mellitus, cancer or cardiovascular diseases and environmental factors such as smoking cigarettes or stress [1,2,5,6,7,8,9]. The standard treatment is to control the infection: removing the plaque by the professional supra and subgingival debridement and by rigorous home dental care [2,10] in association with the intervention on the other factors which was eventually included, such as controlling diabetes or stopping smoking [10].

The beginning of the advance of the plaque is directed apically, causing the alveolar bone resorption [11] which manifests as bone defects, which can eventually lead to tooth loss in advanced cases.

The bone defect that is caused by periodontitis should be treated to reconstruct the structure and function of periodontium [12]. Specifically, after adequate active therapy, when deep pockets (≥6 mm) and intra-bony defects still residue, there is the indication to perform access periodontal surgery [10]. For deep pockets with intra-bony defects of 3 mm deep or deeper, periodontal regenerative surgery is recommended as a treatment [10,13]. By employing periodontal regenerative surgery, we consider the regeneration of the cementum, periodontal ligament, and alveolar bone, which results in the resolution of the intra-bony defect and the gain of clinical attachment [12,14,15]. Currently, regenerative periodontal therapy is performed by eliminating the microbial pathogen and applying biomaterials in the intra-bony defects that work to make wound healing possible. Even though we have a lot of biomaterials and growth factors available, their clinical application has shown that their efficacy is still controversial [16,17]. Moreover, periodontal tissue regeneration via a process that is consistent with the natural tissue formation process has not yet been achieved. For these reasons, periodontal regeneration is still a challenge. Periodontal tissue develops from neural crest cells (NCCs) that reach the cranial region, becoming neural crest derived-mesenchymal cells or ectodermal mesenchymal cells and from their proliferation and differentiation, dental organs and periodontium arise [18]. During the last years, stem cells have been investigated and have provided promising results for tissue repair, regeneration, as well as for the treatment of various diseases [19,20,21,22]. Mesenchymal stem cells (MSCs) have attracted particular concern based on their contributions to tissue cell turnover and response to tissue damage [23]. MSCs are multipotent stem cells; they can differentiate into a variety of tissues, including cartilage, bone, muscle, heart and blood cells. They are important in wound healing, growth, and the replacement of cells that are lost daily through exfoliation or in pathological circumstances [24]. Among the mesenchymal stem cells (MSCs) are the bone marrow-derived MSCs and the dental-derived MSCs group, which includes periodontal ligament stem cells, dental pulp stem cells, stem cells from human-exfoliated deciduous teeth, stem cells from apical papilla and dental follicle precursor cells [23]. The aim of this review is to analyze the clinical utility of the stem cell populations that have been applied for periodontal regeneration, including the bone marrow-derived mesenchymal stem cells and the dental-derived MSCs.

## 2. Bone Marrow-Derived Mesenchymal Stem Cells and Dental-Derived Mesenchymal Stem Cells

**Figure 1:**
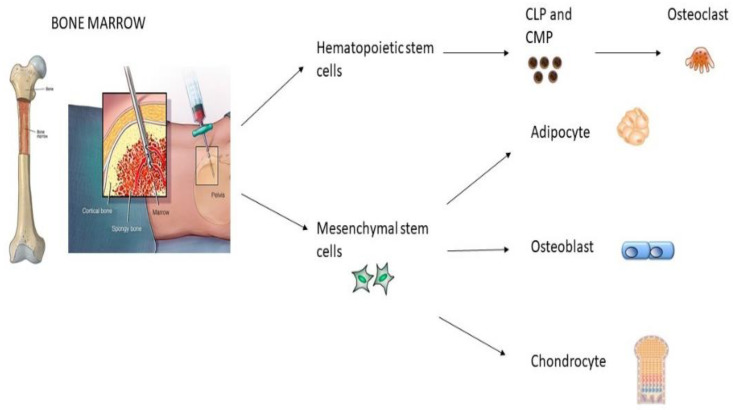
Isolation and differentiation of mesenchymal stem cells from bone marrow.

The conventional isolation of MSCs is based on their typical ability to adhere to plastic surfaces, the identification of specific markers, such as CD105, CD73 and CD90 and their multipotent differentiation potential, that is to say that MSC populations must give rise to at least three cell lineages: osteogenic, chondrogenic and adipogenic ones under standard in vitro differentiation conditions [25] (Figure 1).

BM-MSCs are the most important and a frequent source of isolation of MSCs and have shown good results in regenerative medicine. Some markers characterize BM-MSCs, so it is easy to distinguish them from other cells inside the bone marrow. Those markers are specific antigenic surface proteins such as CD44, CD71, CD90, CD105, CD120a, CD124, CD166, Flt-3 and Kit ligands [26] (Table 1). Their differentiation depends on the biological niche of the tissue, cytokine proteins and specific growth factors [24]. They also have an immunomodulatory role [27].

**Figure 2:**
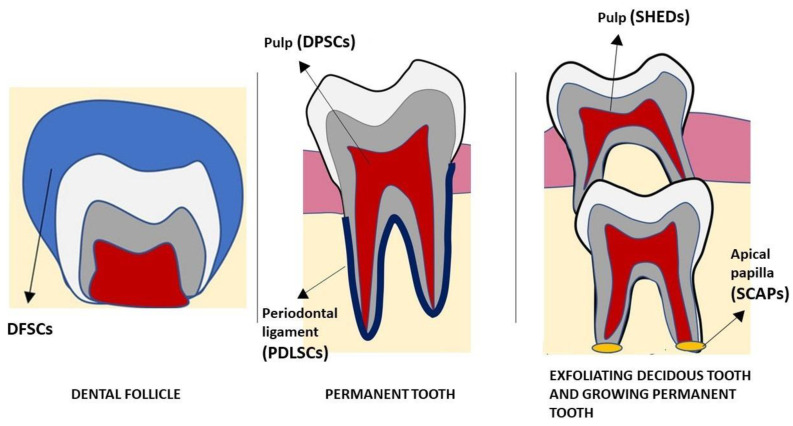
Dental tissues where dental derived stem cells can be harvested. The dental follicle is the niche of DFSCs, the permanent tooth houses the following mesenchymal stem cells: DPSCs in the pulp; PDLSCs in the periodontal ligament; SCAPs in the apical papilla. The pulp of exfoliated deciduous teeth house SHEDs.

**Figure 3:**
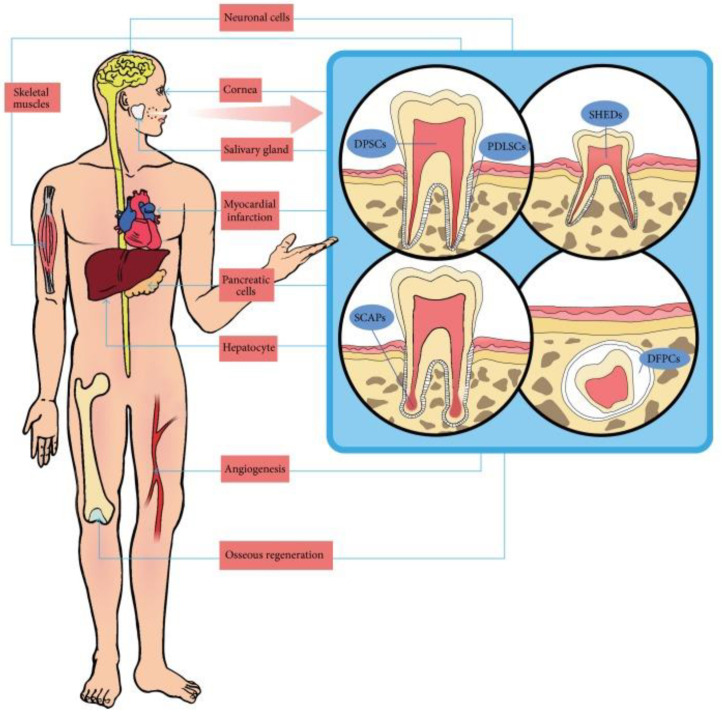
The multipotent potential of dental-derived stem cells. Reprinted/adapted with permission from Park et al. [41] under creative common license.

DMSCs are ectodermal mesenchymal stem cells, which are derived from NCCs, that give rise to dental and periodontal tissues. Depending on the harvest site, they are divided into periodontal ligament stem cells (PDLSCs), dental pulp stem cells (DPSCs), stem cells from human-exfoliated deciduous teeth (SHEDs), stem cells from apical papilla (SCAPs) and dental follicle precursor cells (DFSCs) (Figure 2). DMSCs have a similar multipotent potential as BM-MSCs do, including the possibility to differentiate into odontoblasts, cementoblasts, osteoblasts, chondrocytes, myocytes, epithelial cells, neural cells, hepatocytes, pancreatic cells and adipocytes (Figure 3). They also express the typical mesenchymal markers, such as BM-MSCs do. In addition, it has been shown that DMSCs have an immunomodulatory role, regulating their surrounding microenvironment [37,38].

The immunomodulatory role that BM-MSCs and DMSCs share is very important in periodontal regeneration, considering the microbial etiology of periodontal disease, thus, this property could be very useful in the control of the infection.

To achieve periodontal regeneration by tissue engineering the combination of cells, scaffolds and growth factors have been demonstrated to be efficient. The appropriate progenitor cells differentiate into the mature tissue-forming phenotypes, and their differentiation is stimulated by appropriate signals, which are also essential for favoring the neo-angiogenesis. The scaffold is needed to support and facilitate the cell colonization, migration, growth and differentiation and to provide lost mechanical strength to the damaged site [25].

In addition, recent studies have also shown that new scaffold-free approaches that are based on cell sheets only and are consistent with the usage of recombinant grown factors could be promising in periodontal regeneration [18]. A scaffold-free approach could be a good alternative in periodontal regeneration because it would bypass the typical problems of artificial scaffolds such as biocompatibility, infection or inflammation. Takewaki et al. [39] suggested a promising way to promote periodontal regeneration without an artificial scaffold. They made clumps of an MSC/extracellular matrix (ECM) complex (C-MSC), which consisted of cells sheets that were rolled up and self-produced ECM. Then, they grafted the C-MSCs into furcation III defects in beagle dogs and observed a great response in terms of periodontal regeneration. The limitation of this study is that the dimensions of a furcation III defect are not comparable between beagle dogs and humans. In fact, a furcation III defect in humans could be larger than the beagle dog one was, meaning that it could require larger quantity of MSCs and probably a scaffold too.

Since there is no standardized clinical application of stem cells in periodontal regeneration, and the most critical factor is to identify the most appropriate stem cell population, there is a need to investigate more. Most of the literature of the pre-clinal studies in vivo in animals and in vitro, and recently, a systematic review showed different results on the clinical appliance of stem cells in humans in terms of the reduction and gain of clinical attachment. It has been demonstrated that dental-derived stem cells have given better results than BM-MSCs have in periodontal regeneration [40].

## 3. Bone Marrow-Derived Mesenchymal Stem Cells

Several studies have investigated and demonstrated the potential of periodontal regeneration by using bone marrow-derived stem cells BMSCs [42,43,44,45]. There are different sources to obtain them: bone marrow aspirate BMA [46], bone marrow aspirate concentrates (BMAC) [47,48,49] or cultured and isolated bone marrow stem cells (BM-MSCs) [42,43,45,49,50,51,52,53]. Most of the literature is about the pre-clinical studies on the effects of BM-MSCs in bone, cementum and periodontal ligament regeneration. These studies have shown good results in terms of bone regeneration, for example, after a tooth extraction, the use of BM-MSCs has induced an increase in the local bone density in the subperiosteal buccal alveolar bone surface at the tooth extraction site [54]. Other studies have shown the induction of cementum formation in fenestration defects [49,50,51]. BMAC represents another source of bone marrow-derived stem cells. The literature has not investigated BMAC use often, but it has been demonstrated to be a good choice when regenerating different bone sites [48,49,55,56]. A recent study [57] has compared the use of BM-MSCs and BMAC to treat fenestration bone defects in rats. The results showed the major regeneration efficacy of the BM-MSCs over BMAC. In fact, the BM-MSCs were associated with higher mature bone formation in the early stages than the spontaneous healing and BMAC treatments (*p* = 0.0241 and *p* = 0.0143, respectively) and significantly more cementum-like tissue formation (*p* < 0.0001) with the new insertion of fibers which occurred within 30 days. BMAC were less efficient than BM-MSCs were, but they were better than spontaneous healing was, in fact, they guaranteed a higher bone volume in 30 days, which spontaneous healing did not do (*p* < 0.0001) by enhancing the osteoblastic lineage commitment maturation, with higher levels of osteopontin (*p* = 0.0013) [57]. Even though BM-MSCs are associated with best results, it is important to highlight the difficulties in using them. These are due to the expensive and difficult process of the isolation, characterization, and identification of the appropriate ex vivo culture before their reimplantation and the necessity to use to highly sophisticated laboratories [58,59]. On the other hand, BMAC are very simple to obtain and use and less expensive than BM-MSCs are. As is it mentioned above, there is a lot of pre-clinical research lacking studies that show the impact of BM-MSCs in human periodontal regeneration. Recently, a randomized trial [60] has focused on the usage of autologous BM-MSCs in humans to assess the efficacy, safety and associated benefits of treating infrabony periodontal defects. In this study, twenty-seven infrabony defects were treated and they were randomized in the following three groups: group A, which were treated with autologous alveolar BM-MSCs in combination with a collagen scaffold and with autologous fibrin/platelet lysate (aFPL), group B, which were treated with just the collagen scaffold and the group C, in which a Minimal Access Flap surgery was executed. All of the groups were associated with similar clinical and radiographic outcomes, even if the radiographic bone fillings were inferior in group B. The results showed that periodontal tissue engineering with BM-MSCs is a promising therapeutic option when one is assessing its benefits and its clinical safety.

BM-MSCs have the following advantages: they can be used for allogenic transplantation since they have different surface antigens compared to the mature cells of the tissue; they can be isolated from the patient just before the cell transplantation; they do not cause any immune rejection like other allogenic biomaterials do; they can be expanded in a culture to produce more cells for craniofacial and dental tissue engineering purposes. They also have some disadvantages: they require an invasive process, the rate of proliferation and differentiation reduces with age, a limited amount of them are accessible and there is probability of the destruction of the donor site and low self-renewal [24].

## 4. Dental-Derived Mesenchymal Stem Cells

### 4.1. Periodontal Stem Cells

Periodontal ligament presents different types of cells: cementoblasts, osteoblasts, fibroblasts, myofibroblasts, endothelial cells, nerve cells and epithelial cells. A population of “progenitor cells”, which are organized around the blood vessels, exhibits some of the typical cytological features of stem cells, such as a small size, a responsiveness to stimulating factors and a slow cycle time [61]. Thanks to Melcher in 1985, the concept of the presence of stem cells in periodontal tissues was proposed [62], and then, the evidence came from the in vivo and histological studies of McCulloch and coworkers [61,63,64,65].

PDLSCs can be isolated from teeth at any developmental stage, health status condition and donor age. Furthermore, a non-enzymatic digestion method, named an explant or outgrowth technique, is a suitable protocol for PDLSCs isolation [66].

Periodontal ligament stem cell transplantation into periodontal defects has been demonstrated to be able to enhance the periodontal regeneration [67]. They express the following cell markers: CD44, CD73, CD90, CD105, CD106 and CD146, but not the hematopoietic markers such as CD31, CD34 and CD45 [28,29,30] (Table 1). They can differentiate into osteoblasts, odontoblasts, adipocytes, neural cells, cementoblasts and chondroblasts in vitro [68,69,70,71,72]. This property of PDLSCs means that they can provide the generation of periodontal supporting tissues [73]. PDLSCs that are isolated and cultured from extracted teeth may be used to promote the regeneration of periodontal tissues [24]. In some in vivo studies, the PDLSCs were introduced in periodontal defects in immune-compromised mice, and these demonstrated their capacity to create a periodontal-like tissue [24,68].

In addition to the in vitro studies and the in vivo studies in rats, some clinical trials have investigated the clinical impact of PDLSCs in periodontal regeneration in humans. For instance, in a randomized trial [74], a group of patients, which was defined as the cell group, was treated with periodontal stem cells in combination with Bio-Oss. The control group was treated with GTR and Bio-Oss, only. The results demonstrated the clinical safety of the use of stem cells and a great increase in the alveolar bone in both groups, but there were not any statistically significant differences between them to prefer the treatment with periodontal stem cells. Those results suggested that more investigation was needed to start to use stem cells in clinical activity. Later, another clinical trial [75], in which the test group was treated with PDLSCs and an xenogenic bone substitute and the control group was treated with xenogenic bone substitute only, gave almost the same results as the previously cited study. In fact, the test group showed greater a clinical attachment level (CAL) gain (1.44, standard deviation [SD] = 1.87) and probing pocket depth (PPD) reduction (2.33, SD = 1.32) than the control group did (*n* = 10; CAL gain = 0.88, SD = 1.68, and PPD reduction = 2.10, SD = 2.46), but without statistically significant differences. Thus, the safety in the clinical appliance of periodontal stem cells was assured, but it could not still demonstrated their additional benefit in periodontal regeneration.

Nowadays, most of the literature is about the possibility of enhancing the osteogenic property of the periodontal stem cells by in vitro studies, and they lack recent clinical studies of them. It has been seen that photobiomodulation can enhance the stemness and differentiation capacities of the periodontal ligament stem cells, even though there is not a consensus among scientists for the protocol of their application [76]. A recent study [77] has introduced another possible way to promote regeneration: by using the exosomes that are produced and secreted by PDLSCs. The study showed that exosomes that are secreted from healthy PDLSCs can induce osteogenic differentiation in ill PDLSCs in, in vitro and in bone defects in rats that are affected by periodontitis, suggesting that it can be a therapeutical alternative to treating periodontitis.

In conclusion, there is still a gap between the experimental studies and the clinical appliance of PDLSCs. We lack sufficient data to demonstrate that their contribution to periodontal regeneration is essential and better than other therapeutical alternatives are, even though the above cited studies give us great hope for their future use.

### 4.2. Dental Pulp Stem Cells

Dental pulp is a niche housing tissue of neural-crestal-derived stem cells, which were first identified in 2000 [78]. They have the potential to differentiate into osteoblasts, chondrocytes, myocytes, adipocyte, and neurocytes in vitro and in vivo [31] in addition to their ability to differentiate into odontoblasts to facilitate dentine repair [78].

It has been demonstrated that DPSCs have high proliferative, self-renewal, and multi-lineage differentiation potential [79]. All of the above properties make them a good source for tissue engineering and regenerative medicine.

Dental pulp stem cells also express mesenchymal markers such as: CD29, CD44, CD59, CD73, CD90 and CD146, but not hematopoietic markers [31] (Table 1).

Besides several studies that have investigated the possibility of obtaining different types of tissues, such as neuronal tissue, there are also studies about the usage of DPSCs in periodontal regeneration. Sun et al. [80] have discovered that DPSCs preserve their potential even in patients who are affected by aggressive periodontitis, suggesting that the DPSCs from a periodontally affected tooth may be used for that tooth’s treatment. A review by Amghar-Maach et al. [81] analyzed some studies which were conducted in vivo in animals using DPSCs. Even if a scaffold is important to provide the correct 3D growth of the periodontal tissues, only two of the five presented studies in the review used scaffolds in association with DPSCs. The results showed that if no scaffold is used, the volume of regenerated bone is higher when one is using a HGF-DPSC sheet than when one is using a DPSC injection. Moreover, the best results were associated with the usage of HGF-DPSCs in new periodontal ligament formation and new cementum formation too [82,83]. Among the three scaffold-free studies, one of these [84] showed different results, affirming the superiority of PDLSCs over DPSCs in bone regeneration, new periodontal ligament formation and cementum formation. In the reported study in which Bio-Oss was used as a scaffold with DPSCs, the only additional benefit given by the DPSCs was in terms of new cementum formation [85]. Where PLLA/COL/HA or PisPLLA/COL/HA was used as a scaffold, the best results were associated with the group in which the stem cells were not used [86]. The controversy of the results of the benefit of stem cells is suggested to be due to the immune response against the human DPSCs that were used in animals, in fact, studies in which autologous DPSCs were used gave the best results. Another result that this review gave is that periodontal regeneration does not depend only on the DPSCs, but also on some unknown factors that require further investigation.

Since all of the MSCs must be cultured to have an abundant source of them, it is possible that during the passages, their proliferation and osteogenic ability decrease due to senescence. According to Linsha Ma et al. [87], the most resistant group of the analyzed MSCs during aging was the DPSCs. In fact, the DPSCs showed the greatest maintenance of the proliferation rate, osteogenic property, stemness and the lowest cellular senescence. Moreover, they also were resistant to LPS-induced apoptosis, demonstrating that they preserve their capacities even in inflammatory conditions. This suggests that they could be an excellent source in regeneration during inflammation.

The mentioned studies gave great future strength to the possibility of using DPSCs in regeneration, but none of them was about their application in humans. In the literature, there are some studies in humans that give us more information about the DPSCs. For instance, there is a randomized trial by Ferrarotti et al. [88], in which after a year, the test defects that were treated with DPSCs were associated with a significantly larger probing depth (PD) reduction (4.9 mm versus 3.4 mm), clinical attachment level (CAL) gain (4.5 versus 2.9 mm) and bone defect fill (3.9 versus 1.6 mm) than the controls defects were. Hernández et al. [89] studied the regeneration property of the DPSCs in humans, dividing 22 periodontal patients in an experimental group, which was treated with DPSCs and a collagen matrix and a control group, which was treated with the collagen matrix only. The better results were obtained in the experimental group: it was observed that there was a higher bone regeneration, a reduction of the probing depth and a decrease in the proinflammatory interleukins, which demonstrated, one more time, the immunomodulatory role of DPSCs.

Those studies suggest that DPSCs would be a great source for treating periodontitis bone defects.

### 4.3. Stem Cells from Human Deciduous Teeth

Stem cells from human deciduous teeth (SHEDs) were first isolated in 2003. They present embryonic stem cells markers, such as OCT4 and NANOG, and stage-specific embryonic antigens (SSEA-3 and SSEA-4) which can be advantageous in terms of regeneration therapy and tissue engineering because it means that they may be converted in induced pluripotent cells, thus giving rise to a larger amount of tissues; they also have mesenchymal stem cell markers, such as STRO-1 and CD146 [32,33,34] (Table 1). SHEDs can differentiate into different types of cell lineages such as pancreatic beta cells, hepatocytes, neuronal cells, endothelial cells and odontoblasts [90,91,92,93], and since their origin is from neural ectoderm, they might have the potential to differentiate into neuronal cells [41].

In the literature, some experimental studies show the regeneration potential of SHEDs. For example, in the study of Qiao et al. [94], it was observed in mice that the local injection of SHEDs enhanced the alveolar bone regeneration that is associated with a reduction of the probing depth, even if the normal bone level was not completely reached. Moreover, the immunomodulatory effect of SHEDs was detected; in fact, they inhibited the infiltration of inflammatory factors, such as CD4^+^_T_ cells and INF-γ and TNF-α attenuating periodontitis, plus, the group that was treated with the SHEDs injection was associated with a reduction in the osteoclasts formation, thereby reducing the bone resorption. Therefore, this study showed a concept that had already been suggested by other studies [95,96]: the link between the inhibition of INF-γ and TNF-α and the promotion of periodontal regeneration, even though further investigations are required to clarify the details of the mechanism of this. Fu X et al. [97] studied the effects of allogenic stem cells that were isolated from miniature pig deciduous teeth (SPDs) on periodontal regeneration in pigs. The study revealed that the SPDs promoted periodontal regeneration, moreover, inconsistent with PDLSCs data, they contributed to the regeneration of furcation defects that are usually the most difficult to treat.

Some studies [33,98] have discovered that SHEDs have shown more proliferation rates and higher differentiation abilities than BMSCs and DPSCs have. Another advantage in using SHEDs is related to how they are easy to acquire since they come from an organ, which physiologically exfoliates [94,97]. Furthermore, they can be harvested from the exfoliated teeth of a child and used for the treatment of their parents, which is also more acceptable psychologically [97].

### 4.4. Stem Cells from Apical Papilla

The apical papilla is the soft tissue at the apices of developing permanent teeth. It is the precursor tissue of the radicular pulp, and it is separated from the dental pulp by a cell-rich zone that lies in between [99,100,101]. Thus, stem cells from apical papillae (SCAPs) originate from a developing tissue; this emphasizes the fact that distinct stem cells from those that are found in mature tissues can be found in growing tissues [29].

Stem cells from apical papilla (SCAPs) and the other dental mesenchymal stem cells express the MSC-associated markers and are capable of self-renewal, proliferation and multilineage differentiation. They express the following markers: CD13, CD24, CD29, CD44, CD49, CD51, CD56, CD61, CD73, CD90, CD105, CD106, CD166, NOTCH3 and vimentin (Table 1). It is important to highlight that they express CD24, which is not expressed by DPSCs and may be used to distinguish SCAPs from DPSCs [35]. Compared to DPSCs, SCAPs have a higher expression of antiapoptotic protein survivin, a longer telomere length, and a greater telomerase activity that is associated with cellular lifespan and cell proliferation [35,100,102]. On the other hand, compared to the BMMSCs, the SCAPs secrete more chemokines, neurotrophins and proteins that are involved in metabolic processes and transcription [35,103]. It has been reported that SCAPs can differentiate into different types of cells: odontoblasts, osteoblasts, neural cells, adipocytes, chondrocytes and hepatocytes [35]. Regarding the osteogenic differentiation line, SCAPs have revealed to have nearly the same efficacy that BMMSCs have [104]. In contrast, regarding the odonto/osteogenic property, SCAPs and DPSCs have shown to be superior to BMMSCs [99,105]. In terms of periodontal regeneration, Li et al. [106] examined the role of SCAPs in an experimental periodontitis model in miniature pigs. After 12 weeks of treatment, the results were: enhanced bone regeneration, significantly better values of PD and CAL and a better appearance of the gingival tissue when they were compared to the control group. The same group of the study that is mentioned above, plus other scientists, discovered another important detail which is a gene, SFRP2, which when it is overexpressed, improves periodontal regeneration. In fact, in their study [107], which was conducted in pigs with created periodontal defects, there were three groups: the SFRP2-SCAPs group, which was treated with a local injection of SCAPs with SFRP2 being overexpressed, the SCAPs group, which was injected with SCAPs transduced with a vector backbone and the saline group, which was treated with just a saline injection. After 12 weeks of treatment, the group who was associated with the best results in terms of the gingival health, PD, CAL and GR values was the SFRP2-SCAPs group. This new discovery represents a very important thing to consider to predict in terms of the outcomes of periodontal regeneration therapy, which is in fact that SFRP2 may be a target in periodontal tissue engineering if SCAPs are being used.

### 4.5. Dental Follicle Precursor Stem Cells

The dental follicle is a connective tissue that is derived from the ectomesenchyme, that surrounds the enamel organ and the apical papilla of a developing tooth germ. It is involved in the eruption of the correspondent tooth and in the formation of its periodontium since it contains the precursor cells from which periodontal tissues directly originate [108]. Those cells are called dental follicle precursor stem cells (DFSCs), and it has been demonstrated that in a certain induced environment, they can differentiate into osteoblasts, adipocytes, cardiomyocytes, chondrocytes, neurons, hepatocytes, salivary gland cells and ductal cells [36,109,110,111,112,113,114]. DFSCs express mesenchymal stem cell markers (CD105, CD44, CD29, CD73, CD90, CD146, STRO-1, Notch1 and HLAABC) (Table 1), but not hematopoietic stem cell markers (CD34, CD31, CD45, CD117 and CD14) [36].

Since DFCs are involved in the formation of the periodontium, they are the precursor’s cells of PDLCs, thus, DFCs and PDLCs come from different development stages of the same tissue. Their periodontal regeneration potential has been compared, and as progenitor cells, DFCs have showed more pluripotency, heterogeneity, and proliferative ability than PDLCs have. Moreover, from in vivo experiments, periodontal regeneration has been more efficient when one has been using DFC sheets than when one has been using PDLC sheets [109].

Another experimental study in vivo in rats showed the periodontal regeneration ability of the DFSCs. Given that cementogenesis depends on the interplay between Hertwig’s epithelial root sheath (HERS) cells and the DFCs [115], the scientists of this study [116] decided to test the efficacy of the DFC sheets that were co-cultured with HERS cells in periodontal regeneration. The results confirmed that the HERS cells influence the DFSCs differentiation, considering that the DFC sheets exhibit cemento/osteogenesis-like behavior under HERS cells stimulation. Therefore, the periodontal regeneration property of the DFC sheets was assessed, and in fact, a cementum-like and PDL-like formation in vivo by their use of in vivo experiments in rats was observed.

Even if there have been studies [117,118] that demonstrate that periodontal regeneration can be possible using DFSCs without HERS cells, others suggest that HERS cells’ influence should not be ignored. For example, quite recently in 2018, Guo et al. [119] investigated, once again, the HERS role in the periodontal formation from DFSCs. They assessed that HERS cells directly induct the differentiation of dental follicle cells into periodontal tissues, suggesting that the combination of both sources could be useful in periodontal tissue engineering. Another aspect to highlight is that DFSCs and HERS cells are easily accessible sources, in fact, we can obtain them by simple tooth extraction, for example, from impacted wisdom teeth.

In sum, DFSC is a promising source in periodontal tissue engineering, but it still lacks experiments in humans to assess its clinical efficacy.

## 5. Conclusions

In conclusion, mesenchymal stem cells are a promising source to obtain periodontal regeneration. They have the potential to be a great alternative to current adopted therapies, bypassing the limitations that are associated with the artificial biomaterials. By using MSCs, we would achieve not only the regeneration of periodontium via a natural process, but also the potential resolution of the infection thanks to their immunomodulatory role. For this reason, we believe that further investigations in humans are required in order to assess their practical applicability and to revolutionize the clinical approach when it comes to periodontal regeneration.

## Figures and Tables

**Table 1:** Specific markers expressed from the mesenchymal stem cells, their multipotential differentiation, their application in tooth and periodontal regeneration and their immunomodulatory role.

Cell Population	Markers	References	Multipotential Differentiation	Tooth and Periodontal Regeneration	Immunomodulatory
BM-MSCs	CD44, CD71, CD90, CD105, CD120a, CD124, CD166, Flt-3, and Kit ligands	[26]	Osteogenic Odontogenic Adipogenic Chondrogenic Myogenic Neurogenic	Whole tooth Periodontal tissue regeneration	Yes
PDLSCs	CD44, CD73, CD90, CD105, CD106 and CD146	[28,29,30]	Osteo/ Cementogenic Dentinogenic Adipogenic Chondrogenic Neurogenic	Periodontal tissue regeneration	Yes
DPSCs	CD29, CD44, CD59, CD73, CD90 and CD146	[31]	Osteo/Dentinogenic Adipogenic Chondrogenic Myogenic Neurogenic	Dentin–pulp Tooth root Periodontal tissue regeneration	Yes
SHEDs	OCT4 and NANOG; SSEA-3 and SSEA-4; STRO-1 and CD146	[32,33,34]	Osteo/Dentinogenic Adipogenic Chondrogenic Myogenic Neurogenic Hepatogenic Pancreatogenic	Dentin–pulp Tooth root Periodontal tissue regeneration	Yes
SCAPs	CD13, CD24, CD29, CD44, CD49,CD51,CD56, CD61, CD73, CD90, CD105, CD106, CD166, NOTCH3, and vimentin	[35]	Osteo/Dentinogenic Adipogenic Neurogenic	Dentin–pulp Tooth root regeneration Periodontal tissue regeneration (SFRP2 gene overexpressed)	Yes
DFSCs	CD105, CD44, CD29, CD73, CD90, CD146, STRO-1, Notch1, and HLAABC	[36]	Cementogenic Odontogenic Adipogenic Chondrogenic	Tooth root Periodontal tissue regeneration	Yes

## Data Availability

Data are available from the corresponding author upon reasonable request.
